# The Manchester International Consensus Group recommendations for the management of gynecological cancers in Lynch syndrome

**DOI:** 10.1038/s41436-019-0489-y

**Published:** 2019-03-28

**Authors:** Emma J. Crosbie, Neil A. J. Ryan, Mark J. Arends, Tjalling Bosse, John Burn, Joanna M. Cornes, Robin Crawford, Diana Eccles, Ian M. Frayling, Sadaf Ghaem-Maghami, Heather Hampel, Noah D. Kauff, Henry C. Kitchener, Sarah J. Kitson, Ranjit Manchanda, Raymond F. T. McMahon, Kevin J. Monahan, Usha Menon, Pål Møller, Gabriela Möslein, Adam Rosenthal, Peter Sasieni, Mourad W. Seif, Naveena Singh, Pauline Skarrott, Tristan M. Snowsill, Robert Steele, Marc Tischkowitz, Manchester International Consensus Group, Angel Alonso Sanchez, Mark J. Arends, James Bolton, Tjalling Bosse, John Burn, Emma J. Crosbie, David Church, Joanna M. Cornes, Robin Crawford, Karen Donnelly, Diana Eccles, Richard J. Edmondson, D. Gareth Evans, Ian M. Frayling, Sadaf Ghaem-Maghami, Paula Gollop, Selina Goodman, Heather Hampel, Shirley Hodgson, Noah D. Kauff, Henry C. Kitchener, Sarah J. Kitson, Fiona Lalloo, Anne Lowry, Ranjit Manchanda, Raymond F. T. McMahon, Rhona J. McVey, Usha Menon, Tracie Miles, Gabriela Moeslein, Pal Moller, Kevin J. Monahan, Adam Rosenthal, Neil A. J. Ryan, Peter Sasieni, Mourad W. Seif, Pauline Skarrott, Naveena Singh, Tristan M. Snowsill, Robert Steele, Astrid Stormoken, Helen Stringfellow, Marc Tischkowitz, Andrew Wallace, Luciya Whyte, Nafisa Wilkinson, Godfrey Wilson, Jo Wilson, Nick Wood, D. Gareth Evans

**Affiliations:** 1 Division of Cancer Sciences, Faculty of Biology, Medicine and Health, University of Manchester, St Mary’s Hospital, Manchester, UK; 2 Directorate of Gynaecology, St Mary’s Hospital, Manchester University NHS Foundation Trust, Manchester Academic Health Science Centre, Manchester, UK; 3 Prevention Early Detection Theme, NIHR Biomedical Research Centre, The Christie NHS Foundation Trust, Manchester, UK; 4 Division of Evolution and Genomic Medicine, University of Manchester, St Mary’s Hospital, Manchester, UK; 5 Division of Pathology & Centre for Comparative Pathology, Cancer Research UK Edinburgh Centre, Institute of Genetics & Molecular Medicine, University of Edinburgh, Edinburgh, UK; 6 Pathology Department, Leiden University Medical Center, Leiden, the Netherlands; 7 Institute of Genetic Medicine, Newcastle University, Newcastle upon Tyne, UK; 8 Patient Representative, Cambridge, UK; 9 Department of Gynaecological Oncology, Addenbrookes Hospital, Cambridge University Hospitals NHS Foundation Trust, Cambridge, UK; 10 Faculty of Medicine, University of Southampton, University Hospital Southampton, Southampton, UK; 11 Institute of Cancer and Genetics, Cardiff University, Cardiff, UK; 12 Department of Surgery and Cancer, Imperial College, London, UK; 13 Division of Human Genetics, Department of Internal Medicine, The Ohio State University Comprehensive Cancer Center, Columbus, OH USA; 14 Duke Cancer Institute, Duke University Medical Center, Durham, NC USA; 15 Barts Cancer Institute, Queen Mary University of London, London, UK; 16 Department of Histopathology, Manchester University NHS Foundation Trust, Manchester, UK; 17 Chelsea & Westminster Hospital NHS Trust, London, UK; 18 MRC Clinical Trials Unit at UCL, Institute of Clinical Trials and Methodology, London, UK; 19 Department of Tumor Biology, Institute of Cancer Research, The Norwegian Radium Hospital, part of Oslo University Hospital, Oslo, Norway; 20 Research Group Inherited Cancer, Department of Medical Genetics, Oslo University Hospital, Oslo, Norway; 21 Center for Hereditary Tumors, Helios University Hospital Wuppertal, University of Witten-, Herdecke, Germany; 22 Department of Women’s Cancer, UCL EGA Institute for Women’s Health, University College London, London, UK; 23 School of Cancer and Pharmaceutical Sciences, Kings College London, London, UK; 24 Department of Cellular Pathology, Barts Health NHS Trust, London, UK; 25 Lynch Syndrome UK, Linden House, 9/11 Main Street, Ingleton, Carnforth UK; 26 Peninsula Technology Assessment Group (PenTAG), University of Exeter, Exeter, UK; 27 Health Economics Group, University of Exeter, Exeter, UK; 28 Division of Cancer, Medical Research Institute, Ninewells Hospital and Medical School, Dundee, UK; 29 Academic Laboratory of Medical Genetics, University of Cambridge, Cambridge, UK; 30 National Institute for Health Research Cambridge Biomedical Research Centre, Cambridge, UK; 31 Manchester Centre for Genomic Medicine, Manchester University NHS Foundation Trust, Manchester Academic Health Science Centre, Manchester, UK; 32 Hospital of Navarra, Navarra, Spain; 33 University of Edinburgh, Edinburgh, UK; 34 Manchester University NHS Foundation Trust, Manchester, UK; 35 Leiden University Medical Centre, Leiden, The Netherlands; 36 Newcastle University, Newcastle upon Tyne, UK; 37 University of Manchester, Manchester, UK; 38 University of Oxford, Oxford, UK; 39 Cambridge University Hospitals NHS Foundation Trust, Cambridge, UK; 40 University of Southampton, Southampton, UK; 41 Cardiff University, Cardiff, UK; 42 Imperial College London, London, UK; 43 University of Plymouth, Plymouth, UK; 44 The Ohio State University, Columbus, USA; 45 St Georges, University of London, London, UK; 46 Duke University, Durham, USA; 47 University College London Hospital, London, UK; 48 Eve Appeal, London, UK; 49 University of Witten-Herdecke, Witten, Germany; 50 Oslo University Hospital, Oslo, Norway; 51 Kings College London, London, UK; 52 Lynch Syndrome UK, Carnforth, UK; 53 University of Exeter, Exeter, UK; 54 Dundee University, Dundee, UK; 55 Lancashire Teaching Hospitals NHS Foundation Trust, Lancashire, UK; 56 University of Cambridge, Cambridge, UK

**Keywords:** Lynch syndrome, endometrial cancer, screening, surveillance, guidance

## Abstract

**Purpose:**

There are no internationally agreed upon clinical guidelines as to which women with gynecological cancer would benefit from Lynch syndrome screening or how best to manage the risk of gynecological cancer in women with Lynch syndrome. The Manchester International Consensus Group was convened in April 2017 to address this unmet need. The aim of the Group was to develop clear and comprehensive clinical guidance regarding the management of the gynecological sequelae of Lynch syndrome based on existing evidence and expert opinion from medical professionals and patients.

**Methods:**

Stakeholders from Europe and North America worked together over a two-day workshop to achieve consensus on best practice.

**Results:**

Guidance was developed in four key areas: (1) whether women with gynecological cancer should be screened for Lynch syndrome and (2) how this should be done, (3) whether there was a role for gynecological surveillance in women at risk of Lynch syndrome, and (4) what preventive measures should be recommended for women with Lynch syndrome to reduce their risk of gynecological cancer.

**Conclusion:**

This document provides comprehensive clinical guidance that can be referenced by both patients and clinicians so that women with Lynch syndrome can expect and receive appropriate standards of care.

## INTRODUCTION

Lynch syndrome is an autosomal dominantly inherited cancer syndrome including colorectal (CRC), endometrial (EC), and ovarian cancer (OC).^1^ It is caused by pathogenic variants of the DNA mismatch repair (MMR) system genes *MLH1*, *MSH2*, *MSH6*, and *PMS2*, which prevent the correction of acquired errors during DNA synthesis. Gynecological cancers are often the sentinel Lynch syndrome event in women and have an excellent prognosis.^2^ This provides an opportunity to diagnose women before further oncological sequelae affect them or their family. Early diagnosis allows women to be enrolled in cancer surveillance programs and enables cascade testing for at-risk relatives. There is well-documented survival advantage for those with Lynch syndrome who are compliant with CRC surveillance.^3^ Further, early identification of Lynch syndrome can enable the uptake of cancer prevention strategies, including aspirin and risk-reducing surgery.^4,5^ A timely diagnosis may also have cancer prognosis and treatment implications. For example, MMR-deficient tumors are susceptible to immune checkpoint inhibition through PD-1 blockade.^6^ These considerations necessitate guidelines to direct the identification and care of individuals affected by Lynch syndrome. Although CRC clinical guidance is widely available, the same is not true for gynecological cancers (eTable 1). The lack of comprehensive guidance has led to a nonuniform approach to management of women with Lynch syndrome globally. The aim of the Manchester International Consensus Meeting was to provide the first gynecological-specific guidance for the diagnosis, prevention, and surveillance of Lynch syndrome–associated gynecological malignancies.

### The Manchester International Consensus Meeting 2017 for the management of gynecological cancers in Lynch syndrome

The meeting was held on 24–25 April 2017. Fifty stakeholders attended from across Europe and North America, including patients (*n* = 2), patient support group representatives (*n* = 2), gynecological oncology surgeons (*n* = 12), gynecology nurse specialists (*n* = 5), clinical geneticists (*n* = 10), genetic counselors (*n* = 2), medical oncologists (*n* = 1), colorectal surgeons (*n* = 2), gastroenterologists (*n* = 1), histopathologists (*n* = 10), genetic pathologists (*n* = 1), health economists (*n* = 1), and epidemiologists (*n* = 1).

Preparation for the meeting included a systematic review of the literature to identify key papers to inform discussion. A systematic review with meta-analysis was performed to provide a robust estimate of the prevalence of Lynch syndrome in women with endometrial cancer, using the methodology described in our published protocol.^7^ The body of literature identified through this search also enabled informed discussion regarding the comparable utility of MMR immunohistochemistry (IHC), microsatellite instability (MSI), *MLH1* methylation testing, and direct germline sequencing for pathological variants of the *MMR* genes by next-generation sequencing (NGS) for Lynch syndrome testing (eTable 2). An identical search was conducted in parallel, substituting the Medical Subject Heading (MeSH) term “endometrial” with “ovarian.” Further searches to identify evidence for risk-reducing interventions and the clinical effectiveness of gynecological surveillance in Lynch syndrome (eTable 3) were conducted. Key studies were identified by abstract review and graded according to the category of evidence they achieved (Table 1). Potential bias was assessed by two reviewers, and discrepancies settled by a third, as previously described;^7^ those studies with a high likelihood of bias were excluded. Papers identified through these searches were grouped according to the four clinical questions detailed in this document, and sent to the expert assigned as question lead before the meeting.

**Table 1:** Grading of evidence

Grading of evidence		
Category of evidence	Grading of evidence	
	Traditional	Guideline
Meta-analysis of randomized controlled trials	Ia	A
Randomized controlled trials	Ib	A
Well-designed and controlled study without randomization	IIa	B
Well-designed quasi-experimental study	IIb	B
Nonexperimental descriptive study	III	B
Expert opinion	IV	C

At the meeting, day 1 consisted of 11 lectures covering the prevalence of Lynch syndrome and its associated cancer risks, the patient’s perspective, the process of developing clinical guidance, lessons learned from the colorectal community, current diagnostic technologies, and methods of gynecological surveillance. These lectures provided a critical review of the studies identified through the systematic searches described above and had the purpose of providing the consensus group with up-to-date evidence on which to base its recommendations. Participation was encouraged when assessing the quality of the available evidence during the lectures.

The second day rotated delegates through working groups focused on screening for Lynch syndrome in gynecological cancer, diagnostic methods for such screening, the role of risk-reducing surgery and gynecological surveillance. Each group benefited from multidisciplinary health-care professional and lay representation. Topics were debated until an agreed statement could be reached. The precise wording of these statements was decided through careful deliberation until unanimous agreement was confirmed by a show of hands. Once all delegates had rotated through the four focus groups, a final forum enabled group chairpersons to feed back where consensus had been reached. A further show of hands was required for individual statements to reach this consensus document. The document was written and edited by the expert writing group and circulated through all authors until each recommendation was ratified. The levels of recommendation are shown in Table 2.

**Table 2:** Levels of recommendation

Level	Description	Rationale
Strongly recommend	Patients should expect this level of care	Unanimous agreement from the consensus members. Level of evidence is thought to be sufficient to make this recommendation.
Recommend	Care providers and stakeholders should aim to provide this level of care	Unanimous agreement from the consensus members. Level of evidence is supports the recommendation but is not conclusive.
Neutral	Care providers and stakeholders may wish to provide this service	No unanimous agreement and evidence inconclusive.

### Question 1: Should women with gynecological cancer be screened for Lynch syndrome? (Box 1)

One in 279 of the general population carry pathogenic variants in one of the *MMR* genes, of which the vast majority are unaware.^8^ Approximately 1 in 30 CRCs are Lynch syndrome associated.^9^ The proportion of ECs that are Lynch syndrome associated is around 1  in 30, but estimates are based on small studies hampered by methodological limitations. The largest of these (*n* > 300) are shown in eTable 2.

Current UK guidance from the National Institute for Health and Care Excellence (NICE) supports the universal screening of CRC for Lynch syndrome.^10^ Given similar rates of Lynch syndrome in EC and CRC, and the potential to reduce mortality through colorectal surveillance and cascade testing of relatives, the Consensus Group strongly recommends that women with EC should also be screened for Lynch syndrome.

Restricting screening to those with a higher pretest probability of Lynch syndrome (e.g., younger patients) is likely to reduce the resource burden, although at the cost of missing more Lynch syndrome cases. One study found only 25% of IHC MMR-deficient tumors were in women <50 years.^11^ Hampel et al. found 20% of proven Lynch cases presented >60 years.^12^ While older patients may have a lower risk of Lynch syndrome, and less potential to benefit from risk-reducing measures, they may also have younger relatives who could benefit from the identification of family pathogenic variants. Further, targeted screening has its own challenges, particularly that screening is not conducted despite being indicated.

Amsterdam-II and Bethesda criteria are family history–based prediction tools designed to target Lynch syndrome screening in CRC. Extrapolation of these tools to EC has shown specificity of 61% and 49% for Amsterdam-II and Bethesda criteria, respectively.^13^ Newer prediction tools, MMRpredict_1,26_, MMRpro, and PREMM_5_, have increased sensitivity and specificity.^14–16^ However, they rely on accurate family self-reported history being recorded by the clinician. A quality-controlled family history is time consuming, outside the scope of many busy clinical settings, and does not meet the specificity or sensitivity needed for a first-line test to identify *MMR* pathogenic variant carriers,^17^ however, in the absence of tumor material, such prediction models can be useful to guide germline testing.

Restricting Lynch syndrome screening to tumors with certain pathological phenotypes, for example endometrioid or clear cell morphology, tumors of the lower uterine segment, and those with heavy infiltrates of tumor-associated T-lymphocytes, has not been tested prospectively as a means to direct Lynch syndrome screening.^18^ A low body mass index (BMI) increases the likelihood of Lynch syndrome being the underlying cause in EC.^19^ In CRC, restricting screening for Lynch syndrome to certain high-risk pathological features is not sufficiently sensitive.^20^

With regard to Lynch syndrome–associated OC, there is minimal evidence to guide clinical care. A single-center study found 21% of nonserous epithelial OC to be MMR deficient by IHC.^21^ Lynch syndrome–associated OC is predominantly endometrioid, presenting at an earlier age and stage than sporadic OC, with improved survival rates.^22^ Lynch syndrome is found in 7% of women with synchronous EC and OC.^23^ Many professional organizations now recommend testing all epithelial OC patients for *BRCA1/2* pathological variants.^24,25^ Given the similar cumulative risk of OC in Lynch syndrome, testing premenopausal women with epithelial OC for both *BRCA1/2* and Lynch syndrome is appropriate. This is even more persuasive in an era of panel gene testing where there is no additional cost to add more genes.

There is no evidence to support a link between Lynch syndrome and other gynecological cancers, neither myometrial, nor squamous cancers of the vulva, vagina, or cervix, in which the most important etiological driver is persistent infection with high-risk human papillomavirus (HPV). Thus screening for Lynch syndrome in women affected by cancers of the lower genital tract is not recommended, with the exception of (HPV-independent) endocervical adenocarcinomas, given the difficulty of distinguishing them from lower uterine segment endometrial cancers.^26^

Box 1. Consensus recommendations for screening women with gynecological cancer for Lynch syndrome Where resources are available, the Consensus Group strongly recommends universal screening of endometrial cancer for Lynch syndrome (grade B).

Where resources are restricted, the Consensus Group strongly recommends screening for Lynch syndrome in endometrial cancer where

•Women are diagnosed at 60 years of age or younger (grade B).

•Women diagnosed at any age have one or more of the following risk factors: a personal history of metachronous or synchronous Lynch syndrome–associated cancer, a first-degree relative with Lynch syndrome–associated cancer at 60 years of age or younger, pathological features strongly suggestive of a Lynch syndrome–associated cancer (grade B).

The Consensus Group recommends screening for Lynch syndrome in ovarian cancer where

•Women are diagnosed at 50 years of age or younger (grade C).

•Women diagnosed at any age have nonserous and nonmucinous histology (grade C).

### Question 2: How should women with gynecological cancer be screened for Lynch syndrome? (Box 2)

Screening women with gynecological cancer for Lynch syndrome is a multidisciplinary responsibility. Health-care systems require robust procedures for quality-assured tumor testing and communication of results. Tissue analysis to triage women for Lynch syndrome testing highlights the potential of having Lynch syndrome rather than diagnosing it. Furthermore, identifying MMR-defective status provides important  prognostic information and can direct, for example, immunotherapy treatment strategies.^27^ Thus, MMR IHC/MSI testing should form part of standard patient care and prior consent is *not* required. When germline testing is recommended, informed consent should be sought and patients are entitled to receive specialist counseling.

**Fig. 1:**
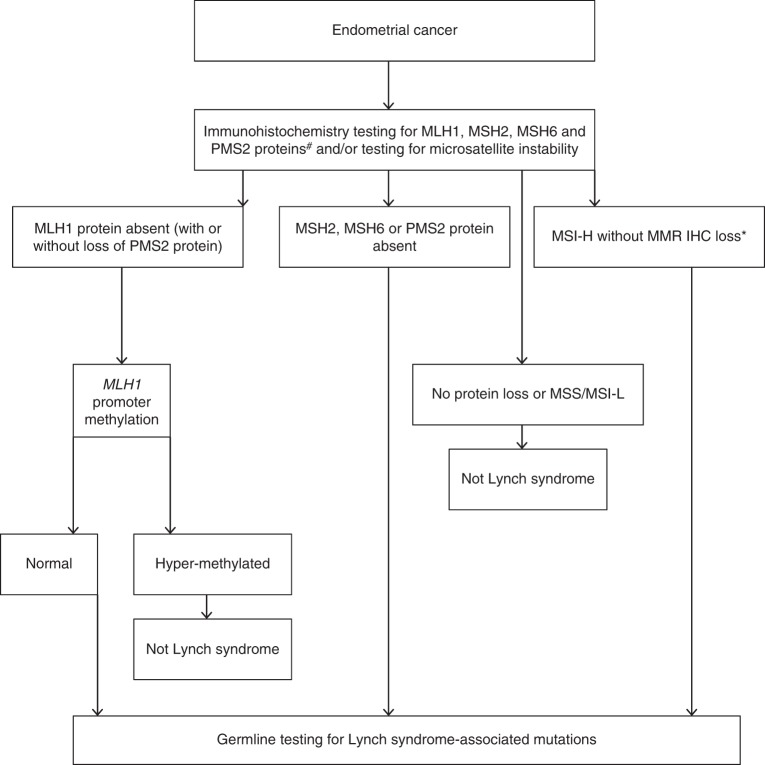
Proposed diagnostic schema of endometrial cancer screening for Lynch syndrome. Consent should be sought from the patient before germline testing. ^#^We recognize the possibility of using a two protein screen using PMS2 and MSH6 initially, however the evidence base is not definitive. *If only microsatellite testing used without immunohistochemistry then all those found to be MSI-H should either be further triaged with methylation testing or undergo direct germline analysis. *IHC* immunohistochemistry, *MSI-H* microsatellite instability high, *MSI-L* microsatellite instability low, *MMR* mismatch repair, *MSS* microsatellite stable.

Four options were explored for the initial screening of tumor samples for Lynch syndrome: MSI, IHC with methylation testing, MSI with IHC and/or methylation testing, and germline NGS (Fig. 1). NGS is the gold standard for identifying somatic pathogenic variants in MMR genes; there remain challenges in working with formalin-fixed paraffin-embedded tumor samples, but performance of NGS on such samples is improving.^28^ It should be noted that exon panel NGS will not identify *MLH1* silencing due to methylation.^29^ Sequencing of *PMS2* is problematic due to the presence of numerous pseudogenes.^30^ NGS is expensive and many hospitals have limited access to it. Thus most studies and current clinical practice employ a screening triage with the use of IHC and/or MSI before germline NGS and large rearrangement testing (eTable 2).

There is good concordance between MSI and IHC analysis.^31,32^ Where there is discordance this may, for example, reflect MSI secondary to *POLE* pathogenic variants, heterogeneity of MMR loss within the tumor, or microsatellite stable (MSS) MSH6 or PMS2 loss.^32,33^ The first is important because *POLE* pathogenic variants have prognostic implications; the last because MSI triage may miss Lynch syndrome cases due to *MSH6* pathogenic variants.^34^ For IHC, sensitivity and specificity range between 86–100% and 48–67%, respectively.^19,35,36^ For MSI, sensitivity and specificity are similar at 77–100% and 38–81% (refs. ^19,35,36^). Where there is a strong family history and where EC/OC presents under the age of 50 years with normal IHC and/or MSI, there is still an argument for definitive NGS.^37^ Investigations should be performed in an agreed stepwise and protocol-driven manner.^10^

IHC analysis has the advantage of identifying the specific protein that has been lost, thus indicating the potentially mutated gene. Furthermore, some pathogenic variants in *MSH6* have been shown to associate with tumor MSS.^38^ The identification of MLH1 protein loss enables methylation analysis, which can exclude women with somatic MLH1 loss from unnecessary NGS.^38^ Methylation-specific polymerase chain reaction (PCR) is simple and cost-effective, however validation work has been almost exclusively in the CRC population.^39,40^ Methylation-specific PCR is not widely available, with only specialist laboratories offering this test. The molecular mechanism for the strong association of *BRAF* variant with CRC harboring somatic MLH1 hypermethylation is incompletely understood but appears to be tissue/tumor-specific; unlike algorithms in use for CRC, BRAF immunohistochemistry or sequencing cannot be used as a proxy for somatic *MLH1* hypermethylation in gynecological cancers, as oncogenic *BRAF* variants occur so rarely in these.^41^ Therefore, moving straight to germline NGS on the basis of IHC MLH1 loss is also an option, although an expensive one.

The MMR proteins form heterodimers, with MLH1 pairing with PMS2 and MSH2 pairing with MSH6. These proteins are unstable in their unpaired state, and while MLH1 and MSH2 can form stable heterodimers with other proteins, PMS2 and MSH6 can only dimerize with MLH1 and MSH2 respectively. It has therefore been proposed that IHC analysis can be performed by testing only two of the four MMR proteins, PMS2 and MSH6, because loss of MLH1 and MSH2 leads to loss of their heterodimer partner (PMS2 and MSH6 respectively).^42^ However, the accuracy of this system is unproven in gynecological cancers.^43^ The interpretation of stained slides requires an experienced senior clinician, and IHC more frequently needs to be repeated due to uninterpretable staining patterns in EC compared with CRC.^44^ Furthermore, the effect of neoadjuvant treatment on tissue analysis for MMR dysfunction has not been defined in EC.^45^ A reporting proforma is shown in eFigure 1. The continuing use of tightly regulated and high quality IHC protocols should be assured by laboratory participation in a national or international external quality assurance scheme that covers both IHC methods and interpretation.

A testing strategy using IHC and MLH1 methylation is likely to be more cost-effective than strategies using MSI testing (because MLH1 methylation must be conducted on all MSI tumors) and strategies not using MLH1 methylation (because methylation testing is cheaper than NGS and excludes a significant proportion of sporadic cases). Documented IHC results can also help interpret NGS results.

Box 2. Consensus recommendations for the methodology used to screen women with gynecological cancer for Lynch syndrome The Consensus Group *strongly recommends* that quality-assured processes for the identification, screening, and reporting of tests for Lynch syndrome are piloted and audited before they are implemented at a local level (grade B).

The Consensus Group *strongly recommends* that tumor MMR or MSI status is used to identify women for germline *MMR* testing. There is no evidence to advocate MSI over MMR immunohistochemistry or vice versa (grade B).

Where MMR immunohistochemistry is performed, the Consensus Group *recommends* testing for all four MMR proteins using formalin-fixed paraffin-embedded biopsy or resected tumor specimens (grade B).

The Consensus Group *strongly recommends* that immunohistochemistry testing is tightly regulated and protocol-driven to ensure interpretable, quality-assured results that can be understood by nonspecialist clinicians (grade C).

Where MLH1 protein loss is identified or initial screen suggests microsatellite instability high (MSI-H), the Consensus Group *recommends* the use of promoter methylation-specific PCR to identify probable cases of *MLH1* silencing and therefore further filter the number of samples requiring NGS, while noting that a small proportion of such cases are due to constitutional methylation or pathogenic variants involving *MLH1* (grade B). BRAF immunohistochemistry or sequencing cannot be used as a proxy for somatic *MLH1* hypermethylation in gynecological cancers (grade B).

For ovarian cancer, the Consensus Group *strongly recommends* somatic NGS on formalin-fixed paraffin-embedded or fresh frozen tumor tissue of nonmucinous invasive epithelial tumors, which should include *BRCA1*/2 and Lynch syndrome genes (grade B/C).

The Consensus Group *strongly recommends* that explicit patient consent is sought before germline testing (grade C).

The Consensus Group *strongly recommends* appropriate referral pathways are established with Clinical Genetics before screening is initiated. This includes those with a positive triage test who are negative for a germline Lynch syndrome pathogenic variant (grade B/C).

Where tumor tests suggest Lynch syndrome but there is no Lynch syndrome–associated pathogenic variant on germline NGS, the Consensus Group *recommends* that clinicians seek to establish the existence of other somatic or germline pathogenic variants, such as biallelic *MuTYH*, *POLE*, and/or double somatic *MMR* pathogenic variants, which may have prognostic implications (grade B).

All recommendations are based on current technology. As this evolves so should clinical practice.

### Question 3: Is there a role for gynecological surveillance in women at risk of Lynch syndrome? (Box 3)

Many female *MMR* pathogenic variant carriers opt to undergo gynecological surveillance in *lieu* of, or whilst awaiting, risk-reducing surgery. The aim of surveillance is to detect premalignant disease or early stage cancer, with the ultimate aim of improving morbidity and mortality from the malignant gynecological  sequelae of Lynch syndrome.

The data relating to gynecological surveillance in Lynch syndrome are generally of low quality, with single-center, retrospective studies predominating (eTable 3). Some studies show benefit and others show no benefit of gynecological surveillance in the early detection of endometrial cancer.^46^ Survival data are limited and mortality data are lacking. However, both preinvasive (atypical hyperplasia) and stage 1 disease have been diagnosed in asymptomatic women undergoing EC surveillance (eTable 3). One study found that women who were not under surveillance were more likely to die from their EC than those in surveillance, although this did not reach statistical significance. In addition, three cancers were “missed” in the surveillance group.^47^ Another found a high proportion of interval ECs in women undergoing surveillance (6/13) (ref. ^48^). It is important to note that EC survival rates in women with Lynch syndrome are extremely good, with 10-year survival of 98% in those undergoing surveillance;^3^ comparative data for LS-EC in women not undergoing surveillance are not available.

The Consensus Group acknowledged that ultrasonography, biopsy, and hysteroscopy could detect EC and premalignant pathological abnormalities. However, there is no evidence that this leads to a stage shift or improved survival in women with Lynch syndrome–associated EC. Furthermore, many patients identified during gynecological surveillance are symptomatic of endometrial pathology (eTable 3). Patient representatives in the Group were strong advocates of gynecological surveillance as a means of regular review and reassurance. Therefore the Consensus Group supported a discussion with individual women as to whether they would wish an annual appointment to undergo detailed symptom inquiry, a rediscussion regarding the option for risk-reducing hysterectomy and bilateral salpingo-oophorectomy and the timing of this, as well as a holistic review of a woman’s contraceptive and fertility needs, and advice regarding cancer risk-reducing behaviors. Women with red flag symptoms of gynecological cancer, including abnormal bleeding, weight loss, bloating, change in bowel habit, recurrent urinary symptoms, and abdominal pain should undergo targeted investigations for gynecological pathology.^49,50^ The Consensus Group was strongly supportive of the need for rapid access facilities being available for women with Lynch syndrome and that suspicious symptoms or signs of malignant gynecological disease should not await routine review in clinic.

Regarding OC, there is currently insufficient evidence that surveillance is of benefit.^46^ Large randomized controlled trials (RCTs) of general population screening for OC through ultrasound scanning (USS) or multimodal screening (CA125 analyzed by an algorithm as a first-line test, followed by reflex USS) have so far failed to demonstrate a statistically significant mortality benefit.^51,52^ However, screening in high-risk *BRCA1/2* pathogenic variant carriers with 4-monthly CA125 analyzed by an algorithm and reflex USS does lead to a stage shift in the disease detected; whether this translates into mortality benefit has yet to be established.^53^ Extrapolation of OC screening research to the LS population is limited by the known biological differences between LS-associated and sporadic/*BRCA*1/2-associated OC.^54^

Whilst there is true equipoise in the literature, high quality research regarding the value of gynecological surveillance in Lynch syndrome could be performed. The options for study design could include a cluster RCT or a centralized repository for routine data collection from local surveillance programs. Studies should be adequately powered to determine whether surveillance picks up earlier disease with benefits for patient outcomes, as well as assessing its psychological impact and cost-effectiveness.

Box 3. Consensus recommendations for gynecological surveillance in women at risk of Lynch syndrome The Consensus Group *strongly recommends* that all women be informed of their *MMR* pathogenic variant-specific risk of gynecological cancer, specifically endometrial and ovarian cancer, interpreted in the context of their family history (grade C).

The Consensus Group *recommends* that women at risk of Lynch syndrome who have not experienced gynecological cancer undergo optional annual review from the age of 25 with an appropriate clinician to discuss red flag symptoms for endometrial and ovarian cancer,^a^ and where contraceptive and fertility needs are raised. A gynecological referral should be made if there is a specific need (grade C).

The Consensus Group does not recommend invasive gynecological surveillance in *MMR* pathogenic variant carriers (grade C), due to insufficient evidence that this improves outcomes over symptom awareness and urgent investigation of red flag symptoms. The Consensus Group *strongly recommends* further research in this area be prioritized by funding bodies. Furthermore, where good quality endometrial cancer surveillance programs already exist, systematic collection of cancer outcomes should be undertaken.

The Consensus Group *strongly recommends* that women participate in routine cervical screening in line with local cervical screening programs (grade A).

^a^Red flag symptoms of gynecological cancer include abnormal bleeding, weight loss, bloating, change in bowel habit, recurrent urinary symptoms, and abdominal pain.^50^

### Question 4: What preventive measures should be recommended in Lynch syndrome to reduce gynecological cancer risk? (Box 4)

Risk-reducing total hysterectomy and bilateral salpingo-oophorectomy prevents gynecological cancer in women at risk of Lynch syndrome.^5^ Surgery is not without risk and potential long-term side effects, however, and preoperative counseling is important. The laparoscopic approach is associated with less postoperative pain, quicker recovery, and improved short-term quality of life, making it the preferred approach in uncomplicated cases, where resources permit.^55^ Surgical menopause follows risk-reducing  oophorectomy in premenopausal women. This is associated with vasomotor symptoms, urogenital dryness and atrophy, reduced sexual function, emotional lability, and cognitive decline, as well as increased risks of osteoporosis, cardiovascular disease and CRC.^56^ Thus prescription of estrogen-only hormone replacement therapy (HRT) until at least natural menopause age (~ 51 years) is strongly recommended to prevent these sequelae. There is some evidence that prior hysterectomy may be associated with greater discomfort during intubation at colonoscopy, and lower cecal intubation rates.^57^ Thus it has been suggested that women undergoing colonoscopic surveillance following hysterectomy undergo specific preprocedure counseling and measures to reduce procedural discomfort.^58^

There are good quality prospective data outlining the cancer risk associated with specific pathogenic variants and the age at which these occur.^59^ A woman’s personal risk should be used to provide individualized counseling regarding the need for risk-reducing surgery and the optimal timing of this. According to the Prospective Lynch Syndrome Database (PLSD; http://www.lscarisk.org), the lifetime risk of EC in women with *MSH2*, *MLH1*, and *MSH6* pathogenic variants is 57%, 43%, and 46%, respectively. The cumulative risk of EC at 40 years of age is 2%, 3%, and 0%, respectively. Lifetime risk for OC in women with *MSH2*, *MLH1*, and *MSH6* pathogenic variants is 17%, 10%, and 13%, respectively. The cumulative risk of OC at 40 years of age is 4%, 3%, and 4%, respectively.^59^ Thus *MSH6* pathogenic variant carriers may consider undergoing risk-reducing surgery after the age of 40 years, while women with pathogenic variants in either *MSH2* or *MLH1* may consider risk-reducing surgery at around 35 years of age assuming their childbearing is complete.^60^ Risk-reducing surgery at 40 years of age is a cost-effective strategy.^61^ The risk of gynecological cancer in *PMS2* carriers is low; however, patient representatives with *PMS2* pathogenic variants felt strongly that they should be offered risk-reducing surgery alongside other women with Lynch syndrome.^59^

There is limited evidence as to how reproductive and lifestyle factors impact on gynecological cancer risk in Lynch syndrome. One study suggested that hormonal influences do modulate cancer risk, however. In addition to combined oral contraceptives, progestin-only methods (pills, injectable, implants, or intrauterine system [IUS]) may protect, but there is little supporting evidence.^62^ The POET trial looked to explore the use of the IUS for the prevention of EC in women with Lynch syndrome. This trial closed due to poor recruitment without results. Another study showed an antiproliferative effect of exogenous progesterone on the endometrium of women with Lynch syndrome, suggesting that these agents could be useful for the chemoprevention of EC.^63^ Exogenous hormones may also protect against CRC.^64^

Aspirin reduces incidence of Lynch syndrome–associated EC and other cancers.^65^ The main toxic effects of aspirin are gastrointestinal. Major gastrointestinal bleeds (those requiring transfusion) are increased in aspirin-takers with an odds ratio (OR) of 1.5–2 (ref. ^66^). However, the absolute rates are small and mainly affect older individuals. Because gastrointestinal toxicity is dose dependent, the optimal dose for cancer risk reduction is being explored through the CaPP3 study of 100 mg, 300 mg, or 600 mg/day (http://www.capp3.org/).

Smoking, alcohol use, and increased BMI may increase the risk of CRC in individuals with Lynch syndrome; however, the impact of lifestyle factors on gynecological cancer risk is unknown. Aspirin may “normalize” EC risk in obese women with Lynch syndrome.^67^ Despite lack of robust evidence, women are advised to eat a healthy diet, avoid obesity, take regular exercise, avoid smoking, only drink alcohol in moderation, and avoid known carcinogens as part of maintaining healthy lifestyles.

Box 4. Consensus recommendations for gynecological cancer risk reduction in Lynch syndrome The Consensus Group *strongly recommends* that risk-reducing total hysterectomy and bilateral salpingo-oophorectomy is offered no earlier than 35–40 years of age following completion of childbearing, in proven *MLH1*, *MSH2*, and *MSH6* pathogenic variant carriers (grade B). A woman’s personal risk should be used to provide individualized counseling regarding optimal timing of the procedure. The strength of evidence is insufficient to strongly recommend risk-reducing surgery in *PMS2* pathogenic variant carriers.

The Consensus Group *strongly recommends* high quality preoperative multidisciplinary counseling supplemented by patient-friendly written information regarding the risks and benefits of risk-reducing surgery (grade C).

The Consensus Group *recommends* preoperative endometrial biopsy and pelvic ultrasound to identify occult gynecological cancer, particularly if a woman is symptomatic (grade C).

The Consensus Group *strongly recommends* that women who are not up-to-date with colorectal surveillance are offered colonoscopy at the time of their risk-reducing surgery (grade B).

The Consensus Group *recommends* that surgery is offered at specialist surgical center, although women at low surgical risk should be able to choose local care (grade C).

The Consensus Group *strongly recommends* all pathological assessment and reporting is carried out at a specialist gynecological pathology center and that the entire endometrium is sampled in the case of prophylactic hysterectomy (grade C).

The Consensus Group *strongly recommends* that women who undergo risk-reducing hysterectomy and removal of their ovaries are offered estrogen-only hormone replacement therapy (HRT) (preferably via the transdermal route) until the natural age of the menopause (age 51 years) or, in consultation with their specialist, until they wish to stop (grade B).

The Consensus Group *recommends* that risk-reducing colorectal and gynecological surgery is carried out at the same time, when indicated and where possible (grade C).^a^

The Consensus Group *recommends* that surgery for colorectal cancer and risk-reducing hysterectomy is carried out at the same time, when indicated and where possible (grade C).

Where risk-reducing surgery has been declined or is not yet appropriate, the Consensus Group *recommends* female carriers of *MMR* pathogenic variants are given the opportunity to discuss their fertility and contraceptive needs with a specialist (grade C).

The Consensus Group *recommends* that the combined oral contraceptive pill is considered for women at risk of Lynch syndrome and wishing contraception because it reduces endometrial and ovarian cancer risk (grade B).

The Consensus Group *strongly recommends* that *MMR* pathogenic variant carriers take aspirin chemoprevention to reduce their risk of colorectal and other cancers (grade A), within the context of a clinical trial (e.g., CaPP3), or through discussion with their doctor.

The Consensus Group *recommends* that women at risk of Lynch syndrome maintain a healthy body mass index (BMI) (grade B).

The Consensus Group *recommends* that women eat a healthy diet, take regular exercise, do not smoke cigarettes, drink alcohol in moderation or not at all, and avoid known carcinogens (e.g., tamoxifen) as part of maintaining a healthy lifestyle (grade B).

^a^Risk-reducing colorectal surgery here refers to an extended colectomy at the time of a colorectal cancer or in the event of any other benign indication for a colonic resection such a multiple (right-sided) recurrent polyps.

## DISCUSSION

This is the first gynecology-focused internationally agreed-upon clinical guideline for the care of women with or at risk of Lynch syndrome.

Our key recommendations are as follows: (1) all stakeholders should be informed of the impact of Lynch syndrome on gynecological cancer risk; (2) systems should be established to screen for Lynch syndrome in women with endometrial cancer; (3) women at risk of Lynch syndrome should be offered risk-reducing hysterectomy with bilateral salpingo-oophorectomy, at a time appropriate to them; and (4) further research is required to establish the value of gynecological cancer surveillance in Lynch syndrome and to explore other key areas where there is currently deficient evidence to define appropriate standards of care. Screening for Lynch syndrome is only recommended if effective management exists to benefit those who screen positive.

The strength of our guidance comes from the broad and expert medical specialty representation that forms our Consensus Group. All relevant stakeholders, including patients and patient advocates, were given equal voice during discussion. Despite different perspectives and expertise, we were able to achieve a consensus view on topics of international importance. By focusing solely on the gynecological aspects of Lynch syndrome, we were able to provide the most comprehensive guidelines yet for the empowerment of both clinicians and patients. This was all achieved without corporate sponsorship.

The major limitation of our work is the lack of robust evidence on which to base our discussions and recommendations. It is hoped that these guidelines will provide the much-needed impetus to inspire researchers and funders to undertake and commission high quality research to fill these gaps in our understanding.

## Supplementary information

Supplemental material
