# Supplementary material for: The Manchester International Consensus Group recommendations for the management of gynecological cancers in Lynch syndrome
Source: Genet Med. 2019 Mar 28;21(10):2390–400. doi: 10.1038/s41436-019-0489-y (PMC6774998; doi:10.1038/s41436-019-0489-y)
Supplement: Supplementary file 1 — Supplemental material [file 41436_2019_489_MOESM1_ESM.docx]

**Supplemental material**

Definitions 2

eTable-1: Gynecological recommendations from current Lynch syndrome guidelines 4

eTable-2: Studies with >300 patients looking at the prevalence of Lynch syndrome in endometrial cancer. 6

eTable-3: Evidence for gynecological surveillance in women with Lynch syndrome. 7

eFigure-1: Recommended Terminology for Reporting Mismatch Repair Protein Immunohistochemistry (MMR IHC) +/- *MLH1* promoter methylation results^a,b,c^ 8

References 11

# Definitions

**Screening** refers to the application of molecular diagnostic methods to women with gynecological cancer in order to diagnose Lynch syndrome.

**Surveillance** refers to the use of diagnostic investigations to detect occult gynecological cancer in Lynch syndrome.

**Pathogenic variant:** In this document, the term pathogenic variant is used instead of ‘mutation’. So, for example, "path_*MSH6*" indicates a pathogenic *MSH6* variant, i.e. causative of Lynch syndrome. Pathogenic variants are either Class 4 or 5 according to the criteria of the InSiGHT MMR gene Variant Interpretation Committee (now recognized as the ClinVar Expert Panel)[https://www.insight-database.org/genes].

**Lynch syndrome** refers to carriers of *MMR* pathogenic variants (in *MLH1*, *MSH2*, *MSH6* or *PMS2*) who have developed cancer. Questions 1 and 2 of the manuscript refer to women with Lynch syndrome-associated endometrial cancer.

**‘At risk of’ Lynch syndrome** refers to proven carriers of *MMR* pathogenic variants who have not yet developed cancer. Questions 3 and 4 of the manuscript refer to carriers of *MMR* pathogenic variants who are at risk of, but have not yet developed, gynecological cancer.

# eTable-1: Gynecological recommendations from current Lynch syndrome guidelines

| **Title** | **Year** | **Screening** | **Surveillance** | **Prophylactic measures** |
| --- | --- | --- | --- | --- |
| NCCN Guidelines Insights Genetic/Familial High-Risk Assessment: Breast and Ovarian. ^1^ | 2019 | No Recommendation given | None endorsed; may offer annual Bx (TVS and CA125 not endorsed) | Consider RRS (H+BSO) at completion of family |
| NCCN Guideline Genetic/Familial High-Risk Assessment: Colorectal Version. ^2^ | 2018 | <50yrs, Known Lynch syndrome family, >5% risk of Lynch syndrome | None endorsed; may offer annual Bx (TVS and CA125 not endorsed) | Consider RRS (H+BSO) at completion of family although insufficient evidence for BSO in *MSH6* and *PMS2* carriers |
| American Gastroenterological Association Institute Guideline on the Diagnosis and Management of Lynch syndrome. ^3^ | 2015 | No Recommendation given | No Recommendation given | No Recommendation given |
| Management of Women with a Genetic Predisposition to Gynaecological Cancers, Scientific Impact Paper No.48. ^4^ | 2015 | IHC if AC or BC met | No Recommendation given | "Benefit not proven". Consider RRS (H+BSO) at completion of family |
| Guidelines on Genetic Evaluation and Management of Lynch Syndrome: A Consensus Statement by the US Multi-Society Task Force on Colorectal Cancer. ^5^ | 2014 | No Recommendation given | Offer annual Bx and TVS | RRS (H+BSO) at 40yrs |
| ACOG Practice Bulletin Number 147: Lynch Syndrome. ^6^ | 2014 | Various options discussed | Annual/biannual Bx from 30-35yrs | Consider hormonal chemoprophylaxis; RRS (H+BSO) at >40yrs |
| Familial risk-colorectal cancer: ESMO Clinical Practice Guideline. ^7^ | 2013 | No Recommendation given | Offer annual Bx, TVS and CA125 | RRS (H+BSO) at 35yrs "may be of benefit" |
| National Society of Genetic Counselors and the Collaborative Group of Americas and Inherited Colorectal Cancer Joint Practice Guideline. ^8^ | 2012 | MSI & IHC if AC or BC met | No Recommendation given | No Recommendation given |
| Revised guidelines for the clinical management of Lynch syndrome (HNPCC): recommendations by a group of European experts/Mallorca Group. ^9^ | 2013 | Universal or <70yrs by MSI OR IHC | Offer annual Bx and TVS from 35-40 | RRS (H+BSO) at 40yrs |
| Report From the Jerusalem Workshop on Lynch syndrome – Hereditary Nonpolyposis Colorectal Cancer. ^10^ | 2010 | No Recommendation given | No Recommendation given | No Recommendation given |
| Risk assessment, genetic testing, and management of Lynch syndrome. ^11^ | 2010 | No Recommendation given | No Recommendation given | No Recommendation given |
| Society of Gynecologic Oncologists Education Committee Statement on Risk Assessment for Inherited Gynecologic Cancer Predispositions. ^12^ | 2007* | No Recommendation given | No Recommendation given | No Recommendation given |
| Recommendations for the Care of Individuals With an Inherited Predisposition to Lynch Syndrome: A Systematic Review. ^13^ | 2006 | No Recommendation given | Annual Bx and TVS from 30-35 | Insufficient evidence for chemoprophylaxis. RRS (H+BSO) at >35yrs |

* A bulletin from the SGO has recommended universal screening of EC (2014), however this has not been formalized into a guideline. Abbreviations: TVS, Trans-vaginal ultrasound; Bx, Endometrial biopsy; RRS, Risk reducing surgery; H, Hysterectomy; BSO, Bilateral Salpingo-oophrectomy; MSI, Microsatellite instability; IHC, Immunohistochemistry; AC, Amsterdam criteria; BC, Bethesda criteria.

# eTable-2: Studies with >300 patients looking at the prevalence of Lynch syndrome in endometrial cancer.

| **Study** | **Patients (n)** |  | |  | |  | | **Tested vs indicated** |
| --- | --- | --- | --- | --- | --- | --- | --- | --- |
|  |  | **MMR protein loss by IHC** | | **MSI-H phenotype** | | **Proven germline pathological variant** | |  |
|  |  | **% loss** | **95 % CI** | **%** | **95 % CI** | **%** | **95 % CI** |  |
| Backes 2011^14^ | 385 | 12.2 | 9.2- 16.0 | NA | NA | NA | NA | NA |
| Batte 2014^15^ | 579 | 7.9 | 6.0-10.1 | NA | NA | 2.1 | 1.2-3.5 | 15 of 45 |
| Bruegl 2014^16^ | 412 | 28.6 | 24.4- 33.3 | NA | NA | NA | NA | NA |
| Buchanan 2014^17^ | 702 | 24 | 22-50 | NA | NA | 3.3 | 1.1-12.3 | 158 vs 170 |
| Buttin 2004^18^ | 413 | NA | NA | 19.1 | 15.6-23.2 | NA | NA | NA |
| Cook 2013#^19^ | 480 | NA | NA | 26.8 | 23.0-31.1 | NA | NA | NA |
| Goodfellow 2015*^20^ | 1002 | 35.9 | 33.0-39.0 | 32.4 | 29.6-35.4 | 1.9 | 1.2-2.9 | 47 of 107 |
| Hampel 2006^21^ | 543 | NA | NA | 21.7 | 18.4-25.5 | 1.8 | 0.9-3.4 | All |
| Joehlin-Price 2014^22^ | 1054 | 24.4 | 27.1-27.1 | NA | NA | NA | NA | NA |
| Kato 2016^23^ | 360 | 2.2 | 1.0- 4.5 | NA | NA | NA | NA | NA |
| Mills 2014^24^ | 604 | 22.6 | 19.4-26.2 | NA | NA | 2.8 | 1.7-4.5 | 21 of 40 |
| Resnick 2010^25^ | 477 | 31.9 | 27.8-36.3 | NA | NA | NA | NA | NA |
| Ring 2016^26^ | 381 | NA | NA | NA | NA | 5.8 | 3.7-8.7 | All |
| **Overall** | 5832 | 21.2 | 18.5-24.1 | 24.3 | 21.7-28.8 | 2.9 | 1.7-4.6 | NA |

Key: *endometrioid tumors only; #<80yrs only. Tested vs indicated refers to the number of patients actually tested for Lynch syndrome using germline NGS vs. the number suspected of Lynch syndrome on the basis of tumour testing

Abbreviations: MMR: mismatch repair; MSI-H: Microsatellite high; NA: Not applicable

# eTable-3: Evidence for gynecological surveillance in Lynch syndrome.

|  | **Modality** | **Interval** | **Pathological variant status** | **Symptomatic** | **AEH detected** | **Cancers detected** | **Stage of cancers detected** | **Cancers Missed** | **Stage of Cancers missed** |
| --- | --- | --- | --- | --- | --- | --- | --- | --- | --- |
| **Cornou 2016 (n=177)^27^** | TVS+Bx+OPH | Annual | Proven LS | NK | NK | 5 EC + 1OC | NK | 0 | NA |
| **Dove-Edwin 2002 (n=222)^28^** | TVS | Annual or Biennial | Mixed | NA | 0 | 0 | NA | 2 EC | 2xI |
| **Gerritzen 2009 (n=100)^29^** | TVS +/- Bx | Annual | Mixed | NK | 4 | 3 EC* + 2 OC | **EC:** Ib, Ic, IIIc **OC:** Ia, IIIc | 0 | NA |
| **Helder-Woolderink 2013 (n=75)^30^** | TVS + CA125 +/-Bx | Annual | Mixed | 0 | 1 | 1 OC | Ia | 0 | NA |
| **Ketabi 2014 (n=871)^31^** | TVS +/- Bx | Various | Mixed | 10 | 3 | 7 EC + 1 OC | **EC:** Ia, 2x Ib, 2xIc, IV, 1xNK **OC:** IIb | 2 AEH, 6 EC, 3 OC | **EC:** 3xIb, II, IIc, IIIc **OC:** 2x1c, IIIc |
| **Le ́curu 2008 (n=57)^32^** | TVS+CA125+Bx+OPH | Annual | Mixed | 2 | 0 | 2 EC | NK | 0 | NA |
| **Manchanda 2012 (n=41)^33^** | TVS+Bx+OPH | Annual | Mixed | 2 | 1 | 3 EC* | **EC**: 3xIa | 0 | NA |
| **Nebgen 2014 (n=55)^34^** | TVS+Bx^ | Annual or Biennial | Mixed | 0 | 2 | 1 EC | Ia | 0 | NA |
| **Renkonen-Sinisalo 2007 (n=175)^35^** | Various | 2-3 years | Proven LS | NK | 4 | 11 EC | **EC:** 5xIa, 4xIb,IIb IIIa, | 4 OC | 3x I & 1x III |
| **Rijcken 2003 (n=41)^36^** | TVS + CA125 +/- Bx | Annual | Mixed | 0 | 3 | 0 | NA | 1 EC | 1B |
| **Rosenthal 2013 (n=95)^37^** | TVS and CA125 | Annual | Mixed | 0 | NK | 3 OC (EC not reported) | **OC:** 1a, 2x1c | 0 | NA |
| **Stuckless 2013 (n=54)^38^** | TVS or Bx or CA125 | Annual or Biennial | MSH2 only | NK | 0 | 5 EC 1 OC | **EC:** 4xIa, IIIa **OC:** IIc | 4 EC 4 OC | **EC:** 2x Ia, Ib, 1 NK **OC:** Ia, IIb, IIc, 1 NK |
| **Tzortzatos 2015 (n=45)^39^** | Various | Annual | Proven LS | 0 | 2 | 3 EC + 2 OC | **EC:** 2xII 1xIa **OC:** 2xIa | 4 EC# | Ia, 2x Ib, II |

*One EC on incident screen, ^ Colonoscopy was carried out concordantly, # 4 AEH/EC found at RRS despite -ve TVS **Abbreviations**: AEH, Atypical endometrial hyperplasia; TVS, Trans-vaginal ultrasound; Bx, Endometrial biopsy; OPH, Out patient hysteroscopy; NK, Not known; NA, Not applicable; EC, Endometrial Cancer; OC; Ovarian cancer.

# eFigure-1: Recommended Terminology for Reporting Mismatch Repair Protein Immunohistochemistry (MMR IHC) +/- *MLH1* promoter methylation results^a,b,c^

| **MMR result** | **Recommended report** |
| --- | --- |
| Normal, MLH1, PMS2, MSH2 and MSH6 tested | **MMR IHC Normal:**  The tumor cells show normal nuclear staining for MLH1, PMS2, MSH2 and MSH6.  Conclusion: There is no immunohistochemical evidence of a mismatch repair deficiency*. |
| Normal, only MSH6 and PMS2 tested | **MMR IHC Normal:**  The tumor cells show normal nuclear staining for PMS2 and MSH6.  Conclusion: There is no immunohistochemical evidence of a mismatch repair deficiency*. |
| Abnormal, MSH6 loss | **MMR IHC Abnormal, MSH6 loss:**  The tumor cells show loss of expression of the mismatch repair protein MSH6 (with normal nuclear staining for MLH1, MSH2 and PMS2).  Conclusion: This mismatch repair deficiency is associated with Lynch and related syndromes.  **This patient should be referred to Clinical Genetics services.** |
| Abnormal, PMS2 loss | **MMR IHC Abnormal, PMS2 loss:**  The tumor cells show loss of expression of the mismatch repair protein PMS2 (with normal nuclear staining for MLH1, MSH2 and MSH6).  Conclusion: This mismatch repair deficiency is associated with Lynch and related syndromes.  **This patient should be referred for methylation testing and if normal, refer to Clinical Genetics services.** |
| Abnormal, MSH2 and MSH6 loss | **MMR IHC Abnormal, MSH2 loss:**  The tumor cells show loss of expression of the mismatch repair proteins MSH2 and MSH6 (with normal nuclear staining for MLH1 and PMS2).  Conclusion: This mismatch repair deficiency is associated with Lynch and related syndromes.  **This patient should be referred to Clinical Genetics services.** |
| Abnormal, MLH1 and PMS2 loss, *MLH1* promoter hypermethylation absent | **MMR abnormality, MLH1 loss and *MLH1* Promoter hypermethylation absent:**  The tumor cells show loss of expression of the mismatch repair proteins MLH1 and PMS2 (with normal nuclear staining for MSH2 and MSH6). *MLH1* promoter hypermethylation is not present.  Conclusion: While this mismatch repair deficiency could be sporadic, it is probable that this mismatch repair deficiency is due to Lynch or related syndromes.  **This patient should be referred to Clinical Genetics services.** |
| Abnormal, MLH1 and PMS2 loss, *MLH1* promoter hypermethylation present | **MMR abnormality, MLH1 loss and *MLH1* Promoter Hypermethylation present:**  The tumor cells show loss of expression of the mismatch repair proteins MLH1 and PMS2 (with normal nuclear staining for MSH2 and MSH6). The *MLH1* promoter shows hypermethylation is present in the tumor.  Conclusion: This combination indicates that this mismatch repair deficiency is almost certainly sporadic rather than due to Lynch Syndrome**.**  **This patient does not require referral to Clinical Genetics services*.** |
| Abnormal, MLH1 and PMS2 loss, *MLH1* promoter hypermethylation not tested | **MMR abnormality, MLH1 loss and *MLH1* Promoter hypermethylation not tested:**  The tumor cells show loss of expression of the mismatch repair proteins MLH1 and PMS2 (with normal nuclear staining for MSH2 and MSH6). *MLH1* promoter hypermethylation has not been tested.  Conclusion: This pattern is likely to be sporadic, although it is possible that this mismatch repair deficiency is due to Lynch or related syndromes. **Testing for *MLH1* Promoter hypermethylation is recommended OR this patient may be referred to Clinical Genetics services.** |
| Abnormal, MLH1 and PMS2 loss, *MLH1* promoter hypermethylation pending | **MMR abnormality, MLH1 loss and *MLH1* Promoter Hypermethylation testing results pending:**  The tumor cells show loss of expression of the mismatch repair proteins MLH1 and PMS2 (with normal nuclear staining for MSH2 and MSH6). *MLH1* promoter hypermethylation testing in the tumor has been requested.  Conclusion: This pattern of mismatch repair deficiency may be either sporadic or due to Lynch or related syndromes – the result of testing for *MLH1* promoter hypermethylation will provide further information. A supplementary report will be issued when these results become available. |

^a^For referral laboratories only reporting mismatch repair status the report should include:

Specimen type:

Site of sample:

Diagnosis:

Overall cellularity (biopsy samples only): High/average/low

Percentage neoplastic nuclei in test area for DNA extraction:

^b^Good fixation is important for obtaining reliable and reproducible patterns of MMR expression by IHC and can be evaluated by assessing MMR expression in internal control cells. Pre-operative biopsies are often better fixed than hysterectomy specimens and may be considered as a better sample for MMR IHC testing, depending on availability. MMR IHC should be reported only in the presence of positive internal control cells, such as stromal cells or lymphoid cells, that are immediately adjacent to the tumor cells under analysis; it must be stated if there is no internal control for comparison.

^c^Rare abnormalities of mismatch repair protein expression are not included in this table and these may be reported as free text where present; examples include weak/patchy/cytoplasmic patterns of abnormal MMR expression, subclonal / heterogeneous patterns of MMR staining abnormality, and loss of expression of different combinations of MMR proteins (other than the expected MLH1/PMS2 or MSH2/MSH6 combinations).

*Referral to Clinical Genetics services should be considered despite this result in the presence of a strong family/clinical history.

# References

1. Daly MB, Pilarski R, Berry M, et al. NCCN Guidelines Insights: Genetic/Familial High-Risk Assessment: Breast and Ovarian, Version 3.2019. https://www.nccn.org/professionals/physician_gls/pdf/genetics_screening.pdf Accessed 23.01.2019

2. Gupta S, Provenzale D, Regenbogen SE, et al. NCCN Guidelines Insights: Genetic/Familial High-Risk Assessment: Colorectal, Version 1.2018. https://www.nccn.org/professionals/physician_gls/pdf/genetics_colon.pdf Accessed 23.01.2019

3. Rubenstein JH, Enns R, Heidelbaugh J, et al. American Gastroenterological Association Institute Guideline on the Diagnosis and Management of Lynch Syndrome. *Gastroenterology*. 2015;149(3):777-782. doi:10.1053/j.gastro.2015.07.036.

4. Scientific Impact Paper No. 48: Management of Women with a Genetic Predisposition to Gynaecological Cancers. *Obstet Gynecol*. 2015;17(2):140-140. doi:10.1111/tog.12182.

5. Giardiello FM, Allen JI, Axilbund JE, et al. Guidelines on genetic evaluation and management of Lynch syndrome: a consensus statement by the US Multi-society Task Force on colorectal cancer. *The American Journal of Gastroenterology*. 2014;109(8):1159-1179. doi:10.1038/ajg.2014.186.

6. Committee on Practice Bulletins-Gynecology, Society of Gynecologic Oncology. ACOG Practice Bulletin No. 147: Lynch syndrome. *Obstet Gynecol*. 2014;124(5):1042-1054. doi:10.1097/01.AOG.0000456325.50739.72.

7. Balmaña J, Balaguer F, Cervantes A, Arnold D, ESMO Guidelines Working Group. Familial risk-colorectal cancer: ESMO Clinical Practice Guidelines. *Ann Oncol*. 2013;24 Suppl 6(suppl 6):vi73-vi80. doi:10.1093/annonc/mdt209.

8. Weissman SM, Burt R, Church J, et al. Identification of individuals at risk for Lynch syndrome using targeted evaluations and genetic testing: National Society of Genetic Counselors and the Collaborative Group of the Americas on Inherited Colorectal Cancer joint practice guideline. *J Genet Couns*. 2012;21(4):484-493. doi:10.1007/s10897-011-9465-7.

9. Vasen HFA, Blanco I, Aktan-Collan K, et al. Revised guidelines for the clinical management of Lynch syndrome (HNPCC): recommendations by a group of European experts. *Gut*. 2013;62(6):812-823. doi:10.1136/gutjnl-2012-304356.

10. Boland CR, Shike M. Report from the Jerusalem workshop on Lynch syndrome-hereditary nonpolyposis colorectal cancer. *Gastroenterology*. 2010;138(7):2197.e1-.e7. doi:10.1053/j.gastro.2010.04.024.

11. Grover S, Syngal S. Risk assessment, genetic testing, and management of Lynch syndrome. *J Natl Compr Canc Netw*. 2010;8(1):98-105.

12. Lancaster JM, Powell CB, Kauff ND, et al. Society of Gynecologic Oncologists Education Committee statement on risk assessment for inherited gynecologic cancer predispositions. *Gynecol Oncol*. 2007;107(2):159-162. doi:10.1016/j.ygyno.2007.09.031.

13. Lindor NM, Petersen GM, Hadley DW, et al. Recommendations for the Care of Individuals With an Inherited Predisposition to Lynch Syndrome: A Systematic Review. *JAMA*. 2006;296(12):1507-1517. doi:10.1001/jama.296.12.1507.

14. Backes FJ, Mitchell E, Hampel H, Cohn DE. Endometrial cancer patients and compliance with genetic counseling: room for improvement. *Gynecol Oncol*. 2011;123(3):532-536. doi:10.1016/j.ygyno.2011.09.002.

15. Batte BAL, Bruegl AS, Daniels MS, et al. Consequences of universal MSI/IHC in screening endometrial cancer patients for Lynch syndrome. *Gynecol Oncol*. 2014;134(2):319-325. doi:10.1016/j.ygyno.2014.06.009.

16. Bruegl AS, Djordjevic B, Batte B, et al. Evaluation of clinical criteria for the identification of Lynch syndrome among unselected patients with endometrial cancer. *Cancer Prev Res (Phila)*. 2014;7(7):686-697. doi:10.1158/1940-6207.CAPR-13-0359.

17. Buchanan DD, Tan YY, Walsh MD, et al. Tumor Mismatch Repair Immunohistochemistry and DNA MLH1 Methylation Testing of Patients With Endometrial Cancer Diagnosed at Age Younger Than 60 Years Optimizes Triage for Population-Level Germline Mismatch Repair Gene Mutation Testing. *JCO*. 2013;32(2):JCO.2013.51.2129–100. doi:10.1200/JCO.2013.51.2129.

18. Buttin BM, Powell MA, Mutch DG, et al. Increased risk for hereditary nonpolyposis colorectal cancer-associated synchronous and metachronous malignancies in patients with microsatellite instability-positive endometrial carcinoma lacking MLH1 promoter methylation. *Clin Cancer Res*. 2004;10(2):481-490.

19. Cook LS, Nelson HE, Stidley CA, et al. Endometrial cancer and a family history of cancer. *Gynecol Oncol*. 2013;130(2):334-339. doi:10.1016/j.ygyno.2013.04.053.

20. Goodfellow PJ, Billingsley CC, Lankes HA, et al. Combined Microsatellite Instability, MLH1 Methylation Analysis, and Immunohistochemistry for Lynch Syndrome Screening in Endometrial Cancers From GOG210: An NRG Oncology and Gynecologic Oncology Group Study. *J Clin Oncol*. 2015;33(36):4301-4308. doi:10.1200/JCO.2015.63.9518.

21. Hampel H, Frankel W, Panescu J, et al. Screening for Lynch syndrome (hereditary nonpolyposis colorectal cancer) among endometrial cancer patients. *Cancer Res*. 2006;66(15):7810-7817. doi:10.1158/0008-5472.CAN-06-1114.

22. Joehlin-Price AS, Perrino CM, Stephens J, et al. Mismatch repair protein expression in 1049 endometrial carcinomas, associations with body mass index, and other clinicopathologic variables. *Gynecol Oncol*. 2014;133(1):43-47. doi:10.1016/j.ygyno.2014.01.017.

23. Kato A, Sato N, Sugawara T, et al. Isolated Loss of PMS2 Immunohistochemical Expression is Frequently Caused by Heterogenous MLH1 Promoter Hypermethylation in Lynch Syndrome Screening for Endometrial Cancer Patients. *The American Journal of Surgical Pathology*. 2016;40(6):770-776. doi:10.1097/PAS.0000000000000606.

24. Mills AM, Liou S, Ford JM, Berek JS, Pai RK, Longacre TA. Lynch syndrome screening should be considered for all patients with newly diagnosed endometrial cancer. *The American Journal of Surgical Pathology*. 2014;38(11):1501-1509. doi:10.1097/PAS.0000000000000321.

25. Resnick K, Straughn JMJ, Backes F, Hampel H, Matthews KS, Cohn DE. Lynch Syndrome Screening Strategies Among Newly Diagnosed Endometrial Cancer Patients. *Obstet Gynecol*. 2009;114(3):530-536. doi:10.1097/AOG.0b013e3181b11ecc.

26. Ring KL, Bruegl AS, Allen BA, et al. Germline multi-gene hereditary cancer panel testing in an unselected endometrial cancer cohort. *Modern Pathology*. 2016;29(11):1381-1389. doi:10.1038/modpathol.2016.135.

27. Cornou C, Bats AS, Vannieuwenhuyse G, et al. Impact of gynecologic screening in Lynch syndrome. *Gynecol Oncol*. 2016;141(Supplement 1):27. doi:10.1016/j.ygyno.2016.04.094.

28. Dove-Edwin I, Boks D, Goff S, et al. The outcome of endometrial carcinoma surveillance by ultrasound scan in women at risk of hereditary nonpolyposis colorectal carcinoma and familial colorectal carcinoma. *Cancer*. 2002;94(6):1708-1712. doi:10.1002/cncr.10380.

29. Gerritzen LHM, Hoogerbrugge N, Oei ALM, et al. Improvement of endometrial biopsy over transvaginal ultrasound alone for endometrial surveillance in women with Lynch syndrome. *Fam Cancer*. 2009;8(4):391-397. doi:10.1007/s10689-009-9252-x.

30. Helder-Woolderink JM, de Bock GH, Sijmons RH, Hollema H, Mourits MJE. The additional value of endometrial sampling in the early detection of endometrial cancer in women with Lynch syndrome. *Gynecol Oncol*. 2013;131(2):304-308. doi:10.1016/j.ygyno.2013.05.032.

31. Ketabi Z, Gerdes A-M, Mosgaard B, Ladelund S, Bernstein I. The results of gynecologic surveillance in families with hereditary nonpolyposis colorectal cancer. *Gynecol Oncol*. 2014;133(3):526-530. doi:10.1016/j.ygyno.2014.03.012.

32. Lecuru F, Le Frere Belda MA, Bats AS, et al. Performance of office hysteroscopy and endometrial biopsy for detecting endometrial disease in women at risk of human non‐polyposis colon cancer: a prospective study. *International Journal of Gynecological Cancer*. 2008;18(6):1326-1331. doi:10.1111/j.1525-1438.2007.01183.x.

33. Manchanda R, Saridogan E, Abdelraheim A, et al. Annual outpatient hysteroscopy and endometrial sampling (OHES) in HNPCC/Lynch syndrome (LS). *Arch Gynecol Obstet*. 2012;286(6):1555-1562. doi:10.1007/s00404-012-2492-2.

34. Nebgen DR, Lu KH, Rimes S, et al. Combined colonoscopy and endometrial biopsy cancer screening results in women with Lynch syndrome. *Gynecol Oncol*. 2014;135(1):85-89. doi:10.1016/j.ygyno.2014.08.017.

35. Renkonen-Sinisalo L, Bützow R, Leminen A, Lehtovirta P, Mecklin J-P, Järvinen HJ. Surveillance for endometrial cancer in hereditary nonpolyposis colorectal cancer syndrome. *Int J Cancer*. 2007;120(4):821-824. doi:10.1002/ijc.22446.

36. Rijcken FEM, Mourits MJE, Kleibeuker JH, Hollema H, van der Zee AGJ. Gynecologic screening in hereditary nonpolyposis colorectal cancer. *Gynecol Oncol*. 2003;91(1):74-80. doi:10.1016/S0090-8258(03)00371-8.

37. Rosenthal AN, Fraser L, Philpott S, et al. Final results of 4-monthly screening in the UK Familial Ovarian Cancer Screening Study (UKFOCSS Phase 2). *ASCO Meeting Abstracts*. 2013;31(15_suppl):5507.

38. Stuckless S, Green J, Dawson L, et al. Impact of gynecological screening in Lynch syndrome carriers with an MSH2 mutation. *Clin Genet*. 2013;83(4):359-364. doi:10.1111/j.1399-0004.2012.01929.x.

39. Tzortzatos G, Andersson E, Soller M, et al. The gynecological surveillance of women with Lynch syndrome in Sweden. *Gynecol Oncol*. 2015;138(3):717-722.
